# Prevalence and anatomy of radix entomolaris and paramolaris in mandibular molars - A CBCT study at North Western India

**DOI:** 10.6026/973206300213780

**Published:** 2025-10-31

**Authors:** Jaspreet Kaur, D. Naveen Prasath, Navjot Singh Khurana, Jagvinder Singh Mann, Tamanna T, Arpit Aggarwal

**Affiliations:** 1 Department of Conservative Dentistry and Endodontics, Government Dental College and Hospital, Patiala, Punjab, India

**Keywords:** Radix entomolaris, radix paramolaris, mandibular molars, root anatomy

## Abstract

The prevalence and anatomy of Radix Entomolaris and Radix Paramolaris in mandibular molars of Punjab Population using CBCT is of interest. Among 271 scans reviewed, the overall prevalence of radix roots was 4.05%, with RE found in 2.9% and RP in 1.1% of cases. The average root length was 14.3 mm and the mean curvature was 46.2°. RE was more frequently seen in first molars, while RP appeared slightly more in second molars. These findings highlight the need for awareness of such anatomical variations to ensure better outcomes in endodontic procedures.

## Background:

A thorough understanding of root anatomy is fundamental for successful endodontic outcomes. Mandibular molars generally have two roots with three to four canals. Clinicians encounter variations in root anatomy commonly, one of such important variation in mandibular molars is the presence of third root termed "Radix" [1]. This additional root is classified as *radixentomolaris* (RE), located distolingually, or radix paramolaris (RP), located mesiobuccally [2]. These anatomical variants are often associated with specific ethnic backgrounds and demonstrate considerable variation in prevalence across different populations [3]. The presence of supernumerary distolingual root was noted and has since been categorized according to the degree of curvature. Several epidemiological studies, conducted on both living individuals and extracted teeth, have highlighted a higher prevalence of radix among indigenous populations from East Asia and the Arctic regions of North America, with occurrences ranging from 5% to 30%. In contrast, Caucasian populations exhibit this feature around 4%. This variation is thus often regarded as a distinctive trait of Asiatic heritage [4]. Accurate knowledge of the canal morphology, including root length and curvature, plays a crucial role in effective cleaning, shaping and obturation of the canal system, directly influencing the prognosis of the therapy [5, 6]. Missed canals or inadequate instrumentation of aberrant roots can lead to persistent infection and treatment failure [7]. Therefore, it is of interest to assess the prevalence and anatomy of radix entomolaris and radix paramolaris in mandibular first and second molars in North Western Indian population.

## Materials and Methods:

The study was conducted in the Department of Conservative Dentistry and Endodontics, Government Dental College and Hospital, Patiala. Cone-Beam Computed Tomography (CBCT) scans performed in the Department of Oral Medicine and Radiology between April 2024 and April 2025 on patients over 20 years old were reviewed. The analysis focused on mandibular first and second molars with fully developed roots. Patients with atleast one mandibular molar were included in the study. Teeth presenting with calcified canals, periapical lesions, previous endodontic treatment, or existing coronal restorations were included. Scans with high-resolution, appropriate contrast and clear diagnostic quality were selected. CBCT scans were taken by VATECH SYSTEM. Images were analysed using VATECH Software. Presence of extra orifice was confirmed in axial sections and morphology of the radix was assessed in sagittal and coronal section. Scans were analysed by 2 endodontists, under supervision of an oral radiologist. Any additional roots identified were classified into two distinct categories: radix entomolaris and radix paramolaris. The software was used to measure the length and curvature of radix root. Shneider's method was used to measure the angulation of root canal curvature. He classified the canal curvature into straight (<5°), moderately curved (if the angle is 10-20°) and severely curved (if the angle is > 20 degrees). The collected data was subjected to statistical analysis.

## Results:

A total of 271 scans meeting the inclusion criteria, comprising of 197 first molars and 74 second molars were analysed. Out of 271 individuals, 130 were males and 141 were females. The results indicated overall prevalence of radix was 4.05 % among the people who underwent CBCT scan in the Department of Oral Medicine and Radiology, Govt. Dental College and Hospital, Patiala, Punjab, India. The prevalence of RE and RP in patients who had radix root were found to be 2.9 % for RE and 1.1 % for RP (Table 1 - see PDF). The frequency distribution revealed that, total incidence of radix entomolaris in first molars was found to be a 2.58% and amongst second molar was found to be 0.3%. Total incidence of radix paramolaris amongst first molars was found to be 0.3% and amongst second molars was found to be 0.7% (Table 2 - see PDF). In samples with RE and RP, the mean root lengths were 14.4 mm and 14.0 mm respectively, yielding an overall average root length of 14.3 mm (Table 3 - see PDF). The mean root curvatures were 47.5° in teeth with RE and 43.0° in those with RP, resulting in an overall average curvature of 46.2°(Table 4 - see PDF). There was a non-significant difference in distribution of the radix root among male and female on application of chi square test (Table 5 - see PDF).

## Discussion:

Lack of knowledge and failure to manage root canal variations, missed canals and inefficient cleaning and shaping are the most common causes of endodontic failure. Evidence suggests that the prevalence of anatomical anomalies varies significantly across different racial, national and regional populations, underscoring the influence of population-specific traits on dental morphology. Radix roots pose a considerable challenge in endodontic treatment due to their complex morphology [7]. Therefore, accurate identification of these anatomical variations is crucial for successful management [8]. Understanding the prevalence of radix roots within specific populations is equally important, as it aids in anticipating such variations during diagnosis and treatment planning CBCT scanner was used in our study to obtain a better analysis of the presence and morphology of radix. Cone-beam computed tomography (CBCT) offers three-dimensional digital imaging capabilities while providing lower radiation exposure and reduced costs compared to conventional computed tomography (CT) scans [9]. The prevalence of radix varies widely among different ethnic groups and geographic populations [10]. Populations with Mongoloid features-such as Chinese, Eskimo and Native American individuals-have shown the highest reported prevalence, ranging from 5% to as high as 30% of radix entomolaris (RE). In Asian communities, Trautman documented notable variability, reporting radix occurrence rates of 17.2% in Indonesians, 10.5% in Singaporeans, 7.9% among Chinese and 5.3% in the Malay population [11]. In contrast, Ferraz and Pecora identified significantly lower radix prevalence rates in other ethnic groups, such as 5% in Madagascans and 2.6% in Brazilians of African descent. Among European populations, their study reported a 1.6% occurrence in Germans, while Brazilians of mixed ancestry showed a prevalence of 4.2% [12]. Additionally, the same authors noted a range of 1% to 8.2% among individuals of Mongoloid origin within the Malay ethnic subgroup, further emphasizing the influence of ethnicity on root morphology. Rahimi *et al.* conducted a study involving 386 cone-beam computed tomography (CBCT) scans obtained from patients at the Oral and Maxillofacial Radiology Department of Tabriz School of Dentistry in north western Iran [13]. They reported a 3% prevalence of three-rooted mandibular first molars, with a slightly higher occurrence in females (3.53%) compared to males (2.50%).

The study found no statistically significant association between gender and the bilateral presence of these additional roots. Similar to many earlier studies, they did not differentiate between the prevalence of radix entomolaris (RE) and radix paramolaris (RP) in their analysis [13]. In contrast to several previous studies, the present investigation specifically classified the additional roots as either radix entomolaris or radix paramolaris in both mandibular first and second molars, allowing for a more detailed assessment of their prevalence and distribution. Our findings contrast with those reported by Dube *et al.* (2011), who documented a considerably higher prevalence of radix entomolaris (9%) in the Indian population [14]. Our study shows a higher prevalence of radix entomolaris in mandibular first molars compared to mandibular second molars. A recent study by Armenta *et al.* (2024) reports the prevalence of radix entomolaris in Mexican population - 3.4% in mandibular first molars and 1.4% in mandibular second molars [15]. The study done on Turkish population, found that the prevalence of radix entomolaris is more than radix paramolaris in mandibular first molars and prevalence of radix paramolaris is more than radix entomolaris in mandibular second molars. The average length of radix entomolaris was found to be 14.4 mm, while that of radix paramolaris was 14 mm. Rokni *et al.* (2023), who reported mean length of 15.5 mm for radix entomolaris and 14.6 mm for radix paramolaris in a Northern Iranian population [16]. Similar results were observed by Kuzekanani *et al.* (2018), who reported an average length of 13.05 mm for radix paramolaris in first and second molars in an Iranian population [17]. Cone-beam computed tomography images of 1315 participants in Slovenia population also exhibited low supernumerary root frequencies comparable to those of other European samples [18]. Average root curvature of RE and RP was found to be 47.5 degrees and 43 degrees respectively. As per the study done by Rokni *et al.* (2023) [16] the average root curvature of RE is approximately 62.33°, while that of RP is about 49.80°, as reported in a CBCT study conducted in Northern Iran. Current study also found that the curvature of radix entomolaris was significantly greater than that of radix paramolaris (P=0.031) which is in similarity to study done by Biswas *et al.* [19].

## Conclusion:

The present study highlights the prevalence and anatomical variations of radix entomolaris and radix paramolaris in mandibular first and second molars in the north-western Indian (Punjab) population. The findings underscore the importance of recognizing these anatomical variations during endodontic treatment, as their presence can significantly influence the outcome. A thorough knowledge of root canal anatomy and the use of advanced imaging techniques are essential for successful diagnosis and management of such anatomical variations in clinical practice.
